# The Role of the Bone Marrow Stromal Compartment in the Hematopoietic Response to Microbial Infections

**DOI:** 10.3389/fimmu.2016.00689

**Published:** 2017-01-20

**Authors:** César Nombela-Arrieta, Stephan Isringhausen

**Affiliations:** ^1^Hematology, University Hospital and University of Zurich, Zurich, Switzerland

**Keywords:** microenvironment, bone marrow, hematopoiesis, microbial infections, stromal cells, niche

## Abstract

Continuous production of blood cells unfolds within a complex three-dimensional tissue scaffold established by highly organized stromal cell networks of mesenchymal, neural, and vascular origin inside bone marrow (BM) cavities. Collectively, stromal cells have been shown to serve two principal roles; first as primary participants of bone remodeling and metabolism and second as master regulators of different stages of blood cell development and production. Indeed, ample evidence demonstrates that stromal cells can sense and integrate systemic signals to shape hematopoietic responses and that these regulatory mechanisms are subverted in multiple pathologic conditions. Microbial infections are stressors that elicit potent inflammatory reactions and induce substantial alterations of hematopoietic output. Whether the cellular components of the BM stromal microenvironment are targeted by infections and participate in infection-induced hematopoiesis has not been investigated in sufficient detail to date. In this manuscript, we provide a succinct updated overview of the different cell populations that are currently known to form BM stroma. We discuss experimental evidence demonstrating that different stromal components are actively damaged or functionally altered by pathogens and/or ensuing inflammatory signals and review how these effects are known to contribute to the hematologic manifestations observed during infections.

## Introduction

Bone marrow (BM) tissues are home to the continuous, lifelong, and high-throughput production of almost all blood cell lineages for the entire lifetime of adult individuals (1). Thus, the BM is arguably the most dynamic and self-renewing organ in the human body. Extremely rare, marrow-residing, hematopoietic stem cells (HSCs) maintain high rates of mature blood cell generation by continuously differentiating along the different branches of the hematopoietic hierarchy. While doing so, HSCs as well as multipotent and lineage-committed progenitor subsets self-renew, thereby preventing the rapid exhaustion of the system (2–4). Highly sophisticated regulatory mechanisms that operate at different levels of hematopoietic development are required to promote an adequate balance between self-renewal and differentiation in order to ensure that continuous cellular production qualitatively and quantitatively matches the demands of organism (5).

Hematopoiesis unfolds within a defined tissue infrastructure provided by BM stromal cells of non-hematopoietic origin, including a variety of endothelial, mesenchymal, and neural cells. Collectively, the BM stromal framework has emerged as a master regulator of hematopoiesis as well as bone metabolism (6, 7). For instance, signals emanating from a variety of stromal components have proven indispensable for the homeostasis of HSC populations. The regulatory crosstalk between stromal cells and HSCs is hypothesized to occur in so-called HSC niches, which have been studied in greatest detail for their relevance and possible implications in stem cell biology and regenerative medicine (8–10). Nonetheless, the functions of endothelial, mesenchymal, and neural cells are not restricted to HSC regulation but extend throughout the entire BM hematopoietic development and include cellular trafficking, lineage-specific differentiation, tissue structural support, and metabolic regulation (6, 11).

Pathogenic infections significantly disrupt the described homeostatic state of the BM by driving profound alterations in the patterns of lineage production, highly increasing the need and rate of hematopoietic output, and thereby imposing a significant strain in the regenerative capacity of the BM (12). As an example, most bacterial infections have been shown to provoke a rapid switch to the so-called state of emergency hematopoiesis, which leads to a shift toward preferential production and BM egress of myeloid cells (i.e., emergency myelopoiesis) (13, 14). Such conserved responses most likely have evolved to promptly compensate for the massive consumption of immune cells, mostly granulocytes or monocytes that are recruited to infection sites (12). In contrast, certain viral agents are well known to lead to BM suppression and pancytopenia (15). Thus, BM hematopoietic responses are highly pathogen-specific, will vary depending on microbial tropism and host immune responses, and need to be studied in an individualized fashion. Hematopoietic perturbations are generally the consequence of the combined effects of (i) direct pathogenic targeting of relevant hematopoietic and non-hematopoietic cell types, (ii) the action of microbe-associated molecular patterns (MAMPs) through activation of pattern recognition receptors PRRs in a variety of cell subsets, and (iii) the inflammatory cytokines released as part of immune responses destined to control infectious challenges (16).

Numerous recent experimental studies have focused in dissecting how hematopoietic cells in the BM directly respond to microbial infections. HSPCs themselves, given their prominent position at the apex of the hierarchy, represent a unique target for the modulation of hematopoiesis in a global, rapid, and versatile fashion during stress (5, 16–18). Indeed, it was recently demonstrated that HSCs are primary responders to infections as they are directly targeted by MAMPs and multiple inflammatory cytokines, such as IFNα, IFNγ, IL-1, interleukin-6 (IL-6), or TNFα, which distinctively affect their cycling properties, differentiation, repopulating activity, and migration patters (5, 16, 17, 19). Notably, the instructive effects of host and pathogen-derived mediators are not limited to HSCs. Different multipotent and lineage-restricted progenitor subsets have been shown to undergo direct infection by certain intracellular pathogens and/or respond to MAMPs and cytokines, which can eventually trigger deviations or blockage of their normal differentiation potential and lineage output (20–22).

Altogether, it seems clear that the different ways in which hematopoietic elements sense and respond to pathogenic stimuli and ensuing inflammation will critically shape the global BM hematopoietic response during the different phases of a microbial infection. Yet, given its well-documented essential role in the orchestration of steady-state hematopoiesis, the potential contribution of the BM stromal microenvironment to infection-mediated hematopoiesis should not be neglected (18). Whether and how BM-resident stromal cells are targeted by infections still remains poorly defined. Furthermore, to what extent structural and functional changes in BM stroma underlie the observed effects of pathogens in hematopoietic function needs to be further investigated. Notably, experimental evidence gathered in a variety of tissues but specifically in lymphoid organs strongly indicates that in general, stromal cells are not passive bystanders but rather play a very active role in remodeling of organ structure and physiology during stress responses (23, 24). In this article, we provide a brief account of the different stromal subsets forming the BM microenvironment and their regulatory control of hematopoiesis as delineated by recent studies in the murine system and summarize what is known on the pathophysiological responses of these cells to infections. Finally, we revise relevant knowledge derived from clinical practice, which points to a prominent role of BM stromal cells in the hematological manifestations observed in human infectious diseases.

## Cellular Components of BM Stroma

The presence of a rich non-hematopoietic cellular fraction in the BM had been long appreciated and its likely role as a fundamental orchestrator of hematopoiesis anticipated for many years (25). However, its constituents were until recently only vaguely grouped under the term “stroma,” their heterogeneity and functions remaining underappreciated (26). Driven by a number of technical and methodological advances, remarkable progress has now been achieved in the understanding of the composition, phenotypic characterization, developmental origin, anatomy, and crucial functions of BM stromal components. First, an assortment of reporter mouse models in which mesenchymal, vascular, and neural BM populations are genetically targeted and/or fluorescently illuminated in a specific manner have been generated (6, 27). In parallel, protocols that include the enzymatic digestion of BM tissues and permit the extraction of large stromal cells now enable their identification by flow cytometry and prospective isolation *via* cell sorting, for a detailed molecular and functional analysis (28–30). Finally, improved *in situ* or *in vivo* imaging approaches that allow the simultaneous visualization of diverse BM components have been developed and are now being widely employed to understand their three-dimensional microanatomical organization (31–34). Collectively, these new tools are proving invaluable for the mapping of the heterogeneity contained in BM stroma, as well as for the understanding of the ways in which these multiple cell types orchestrate hematopoiesis.

### BM Endothelial Cells (BMECs)

**Figure 1:**
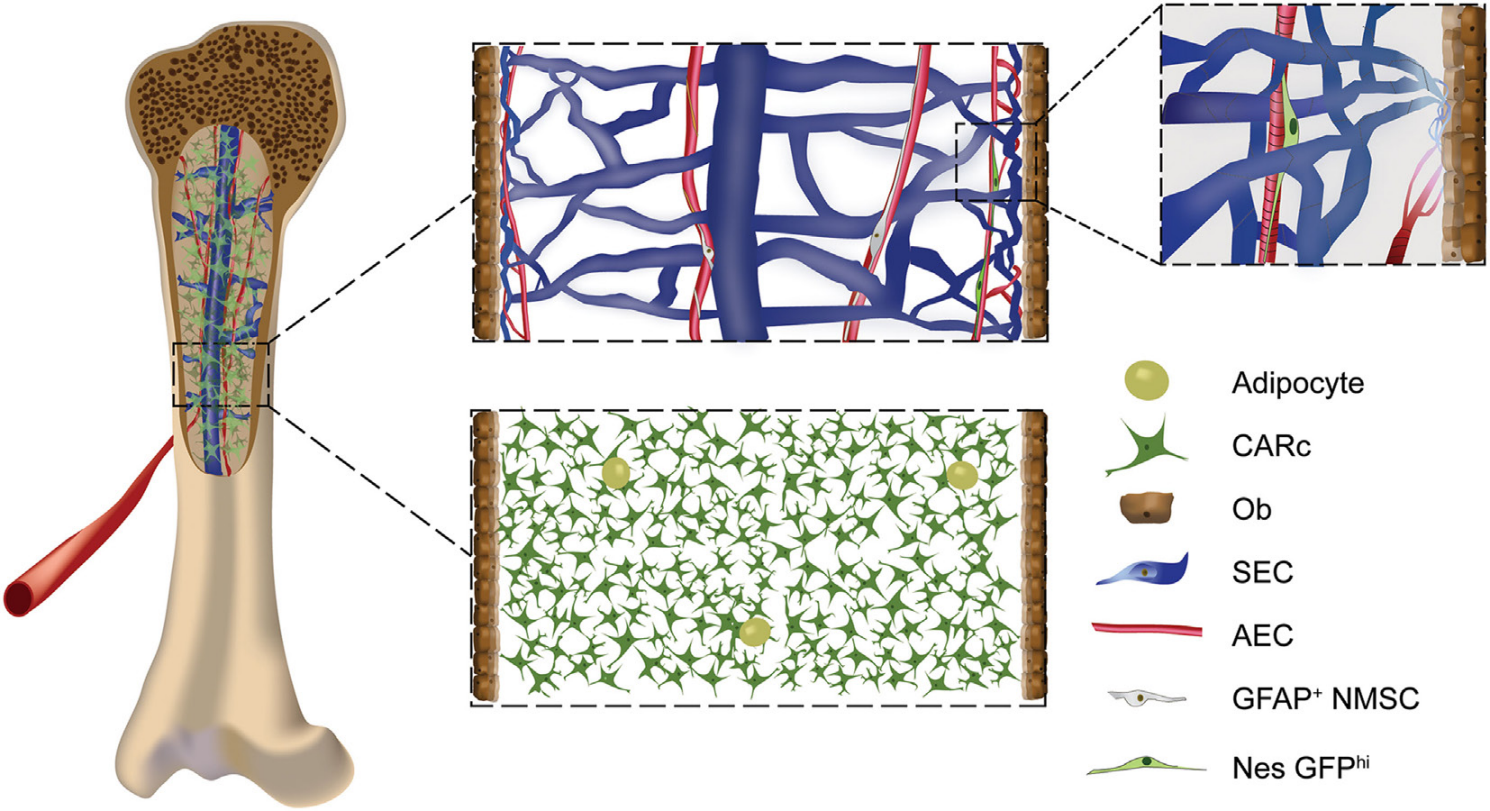
**Cellular components of the stromal compartment of the bone marrow (BM): schematic overview of BM stromal cellular constituents in a mouse femur**. Endothelial cells of arterial (AECs), transitional (type H), and sinusoidal [sinusoidal ECs (SECs)] subtypes form the vascular system of the BM. Densely packed AECs form arteries and arterioles, which connect to type H transitional vessels, that give rise to sinusoids made up by large SECs (upper panel, zoomed-in image). AECs, type H, and SECs have different morphological, phenotypic, and molecular features and have been shown to play specific roles in the regulation of hematopoiesis and osteogenesis. The neural component of BM stroma is formed by rare non-myelinating Schwann cells and adrenergic neurons. The mesenchymal compartment includes progenitor subpopulations such as fibroblastic reticular stromal cells, also termed CXCL12-abundant reticular cells (CARc), and Nes-GFP^hi^ cells (upper panel, zoomed-in image). Mature mesenchymal cells are composed of bone-lining osteoblasts (Obs) and adipocytes.

The BM is perfused by an extremely dense microvascular tree comprised by a variety of BM endothelial cell subtypes, which assemble into distinct vessels that largely differ in morphology, phenotype, and function (30, 31, 35–37). Following the archetypical structure of vascular trees, oxygenated blood enters the BM through arterial vessels, which branch into thinner arterioles. Arterial circulation transitions into large venous structures through a set of transitional vessels (also termed type H) in the proximity of endosteal surfaces (31, 32, 35). In the BM, venous thin-walled vessels construct a dense and highly interconnected network designated as sinusoidal (38) (Figure 1). Arterial vessels display low permeability, shielding the surrounding extravascular space from access to plasma-derived factors (36). Due to their anatomical localization at the interface between BM and bone surfaces, type H vessels critically influence bone remodeling (35). Sinusoids in turn are highly permeable and therefore serve as the main trafficking routes for cells in and out of BM tissues (39, 40).

Different subtypes of BMECs can be isolated and studied based on their specific phenotypic signature (29, 30, 36), the distinct expression of adhesion molecules, and the production of trophic hematopoietic factors, which has proven of relevance for their different biological functions (11, 41). As an example, sinusoidal ECs (SECs) among other angiocrine factors produce high levels of stem cell factor (SCF), CXCL12, and E-selectin, through which they critically participate in the maintenance and quiescence of HSPCs in perisinusoidal niches (42–45). Based on this molecular profile and the spatial interactions they establish, SECs have been further postulated as key regulators in different stages of hematopoietic cell development, such as the maintenance of mature megakaryocytes or the differentiation of immature B cells (7, 40, 46–48). An essential additional task of BMECs is the regulation of tissue regeneration and restoration of HSC homeostasis after extreme injury with ionizing radiation or chemotherapeutic agents, to which the fragile BM hematopoietic compartment is highly sensitive (29).

### BM Mesenchymal Cells

The mesenchymal compartment of the BM is not only engaged in hematopoietic regulation but also it contains a stem cell-based hierarchy required for bone development, homeostatic remodeling, and repair (49). The developmental roadmap by which multipotent mesenchymal stem cells (MSCs) give rise to chondrocytes, osteoblasts and adipocytes *in situ* within the BM is much less clearly defined than the hematopoietic hierarchy. Recent reports have provided new insight into the mesenchymal compartment through the use of novel genetically engineered mouse models to target and trace mesenchymal populations. These studies are gradually uncovering a rather complex scenario (50–52). The nuances of the system have been elegantly and thoroughly reviewed elsewhere and largely exceed the scope of this manuscript (6). For the purpose of this review, it is important to note that a general confusion has historically pervaded the field regarding the use of the term MSCs. Besides referring to the true bona fide skeletal stem cell and mesenchymal progenitors present in native BM tissues, the name MSC was indistinctively employed to designate the product of the *in vitro* clonogenic expansion of the adherent cell fraction isolated from different organs, which featured adipogenic, osteogenic, and chondrogenic potential *in vitro* (49, 53). The latter have been extensively characterized for their immunomodulatory properties *in vitro* and upon transplantation, which may be therapeutically exploited in the context of severe infectious and inflammatory challenge (54). However, in this manuscript, we will exclusively refer to those studies, which have addressed the effects of infections in MSCs or mesenchymal progenitor cells that reside in and can be prospectively isolated from BM tissues.

Mesenchymal stem cells are proposed to give rise *in situ* to a fibroblastic reticular cell network that densely populates BM and holds strong morphological and structural resemblance to stromal lattices found in other lymphoid organs, such as lymph nodes and spleen (23) (Figure 1). BM reticular cells, identified based on their expression of leptin receptor (Lepr), comprise the majority of progenitor cells that generate osteoblasts and adipocytes in steady state, as well as chondrocytes and reticular stroma in the context of bone repair (55–57). Fibroblastic stromal cells abundantly express factors that are absolutely indispensable for the hematopoietic function of the BM (55). For instance, they are the most prominent source of CXCL12 [thus designated as CXCL12-abundant reticular (CAR) cells] and SCF, through which they promote retention and maintenance of HSPCs in the BM (42–44). Beyond their regulation of early hematopoietic progenitors, at least a subset of Lepr^+^ reticular cells additionally secretes interleukin-7 (IL-7) and therefore contributes to different stages of lymphoid lineage development (47, 58, 59). Other less well-characterized subpopulations of mesenchymal origin have been described. These include periarteriolar cells defined in the NestinGFP mouse model by the highest expression of the transgene, and a subset of perivascular cells found mostly in trabecular bone regions, which express NG2 and PDGFRβ (33, 60).

Mature osteoblasts line the inner surfaces of bone and constitute one of the direct mature derivatives of mesenchymal reticular stromal cells (56, 61). Although their primary function is the production and secretion of mineralized matrix needed for continuous remodeling of bone surfaces, osteoblasts have been extensively studied for their regulatory crosstalk with the hematopoietic system. Initial studies pointed to a role of osteoblasts in providing a supportive HSC niche (62, 63). Nonetheless, recent evidence suggests that signals derived from the mature osteoblastic or endosteal niche are rather required for the maintenance of lymphoid progenitors and balanced T and B cell lymphopoiesis (43, 64–67). One of the alternative cellular fates of mesenchymal Lepr^+^ cells is differentiation into adipocytes (55, 56). Marrow adipogenesis is a highly complex phenomenon, the functional implications of which are only superficially understood. In general terms, adipocytes are regarded as negative regulators of hematopoiesis, which emerge mostly in the context of pronounced marrow injury and aging [(68); reviewed in Ref. (69)].

### BM Neural Cells

Cells of neural origin include glial GFAP^+^ non-myelinating Schwann cells (NMSCs) and adrenergic neurons, which are scarcely distributed throughout the BM interstitium (70, 71). NMSCs have been shown to participate in the maintenance of HSC quiescence through production of activated transforming growth factor-β (70). Adrenergic signals relayed by sympathetic nerves most likely play multiple unexplored and complex roles in BM hematopoietic function but have been studied for their control of the constant cellular trafficking in and out of the BM (71, 72). During homeostasis, HSCs are rhythmically released from BM into peripheral circulation in a pattern that is governed by the adrenergic-mediated control of CXCL12 expression levels (71). Likewise, continuous cell recruitment to the BM, which is dependent on cellular adhesive interactions with the microvasculature, oscillates following circadian cycles established by adrenergic signaling (72). Neural-derived signals have been additionally demonstrated to critically contribute to the complete and rapid structural regeneration of BM post-injury (73).

## Infection-Induced Alterations in BM Stromal Microenvironment

**Figure 2:**
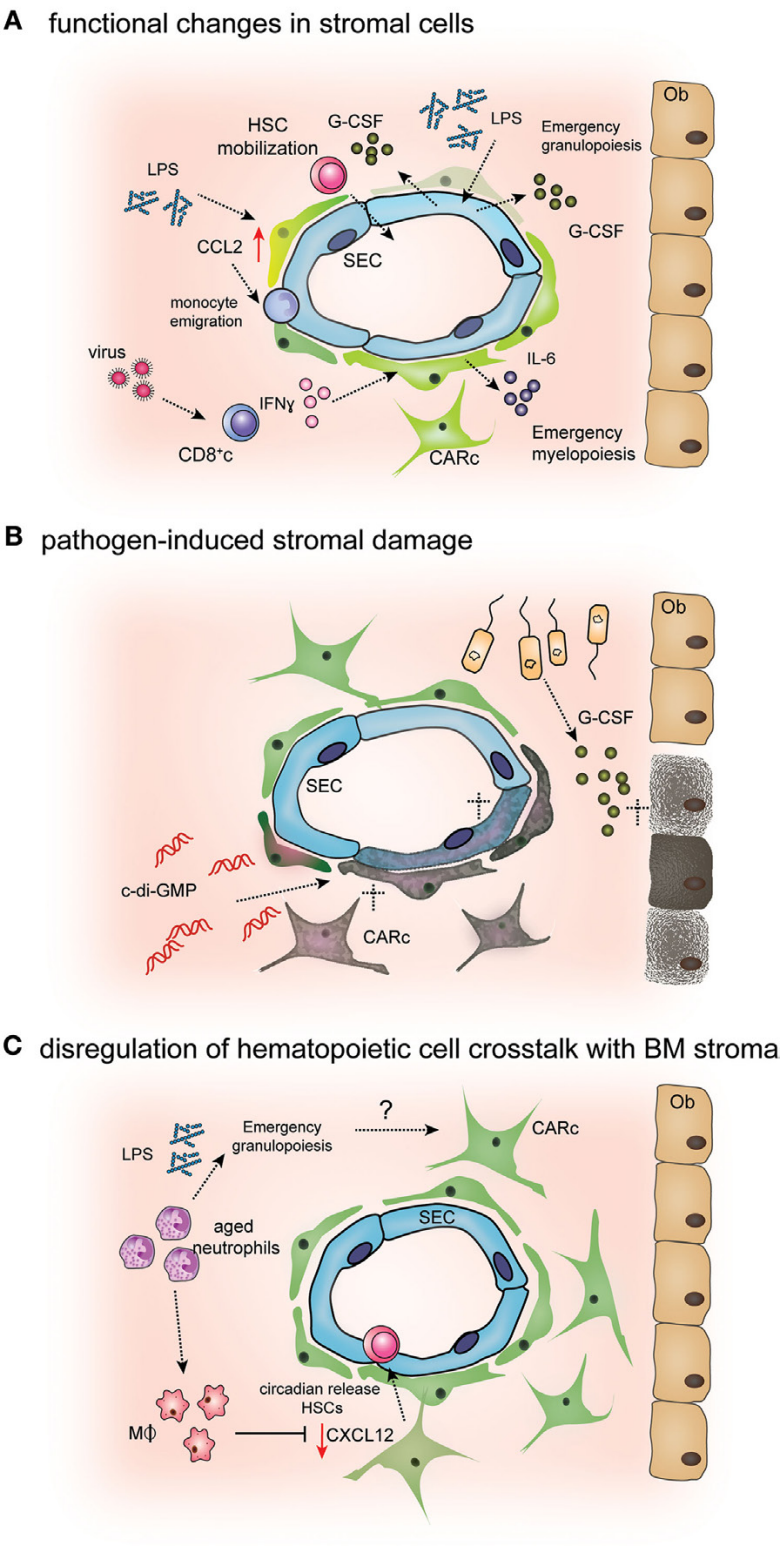
**Mechanisms of pathogen-induced alterations in bone marrow (BM) stromal cells**. **(A)** Endothelial and mesenchymal BM cells sense microbial-derived products or inflammatory cytokines. Lipopolysaccharide (LPS) stimulates endothelial cell [sinusoidal EC (SEC)] secretion of G-CSF, leading to emergency granulopoiesis and HSPC mobilization (76, 90). Secretion of IFNγ by CD8 cells during antiviral responses activates interleukin-6 (IL-6) production by CXCL12-abundant reticular (CAR) cells, which in turn induces biased differentiation of HSPCs toward myeloid lineages (81). Bacterial LPS has also been shown to stimulate CCL2 production by CAR cell, leading to monocyte extravasation from BM into circulation (78). **(B)** Excess levels of pro-inflammatory cytokines or active targeting by pathogens can induce stromal cell death leading to aberrant BM microenvironmental regulation of hematopoiesis. During polibacterial sepsis, osteoblast (Ob) numbers are decreased through the activation of apoptotic programs mediated by G-CSF (75). Administration of the bacterial second messenger c-di-GMP leads to reduced numbers of CXCL12-abundant reticular cells (CARc) and SECs (80). **(C)** Myeloid cells, mainly macrophages and aged neutrophils, modulate CXCL12 production by CAR cells, leading to the cyclic release of hematopoietic stem cells (HSCs) from BM to circulation (91, 92). Given that infections and inflammatory challenges massively change the numbers and frequencies of myeloid cells in BM, it is conceivable that such changes promote alterations in CAR cell functionality.

Although the basic observation that balanced hematopoiesis and consequently BM dynamics are highly reactive to infectious challenges was recognized longtime ago, relatively few experimental studies have addressed how specific pathogenic infections and ensuing inflammatory responses directly impact the composition, structure, and function of BM stroma (Table 1). On the basis of recent investigations in animal models of infections, we here propose three main types of mechanisms by which pathogens may lead to major perturbations of the microenvironmental control of BM function (Figure 2).

**Table 1:** **Reported effects of mouse and human pathogens in bone marrow (BM) stromal cells**.

Type of pathogen	Pathogen/microbial product	BM stromal subset targeted	Hematopoietic response	Mechanism	Reference
**Parasite**	*Toxoplasma gondii*	Mesenchymal stromal cells	Enhanced myeloid output, block in erythropoiesis	Stromal-derived interleukin-6 (IL-6) secretion	(74)

**Bacteria**	Polymicrobial, sepsis model	Osteoblasts	Ablation of common lymphoid progenitors, block in T and B lymphopoiesis	G-CSF-mediated ablation of osteoblasts and decrease in interleukin-7	(75)
	*Escherichia coli* and lipopolysaccharide administration	BM endothelial cells (BMECs) and ECs from other organs	Emergency granulopoiesis	TLR4-mediated and G-CSF-dependent	(76, 77)
			Mobilization of HSPCs to blood and spleen	TLR and NOD-induced G-CSF secretion in non-hematopoietic cell	(76)
	*Listeria monocytogenes*	Mesenchymal osteoprogenitors/CXCL12-abundant reticular (CAR) cells	Monocyte mobilization	Mesenchymal stromal cell expression of CCL2	(78)
	*Mycobacterium tuberculosis* (human)	Mesenchymal stromal cells	Not defined	Direct intracellular infection of mesenchymal stromal cells	(79)
	c-di-GMP (bacterial second messenger)	Osteoprogenitors, CAR cells and BMECs	Decrease in numbers and dysfunction of BM HSCs and extramedullary hematopoiesis	Decrease in BMECs and BM mesenchymal stromal cell populations	(80)

**Virus**	*Lymphocytic choriomeningitis* virus	Mesenchymal stromal cells	Biased differentiation of HSPCs toward myeloid lineage	IL-6 secretion by mesenchymal stromal cells triggered by IFNγ from CD8 T cells	(81)
	MCMV	*In vitro* cultures of mesenchymal stromal cells	Loss of HSPC supportive capacity—potential cause of aplasia	Direct infection and decrease of hematopoietic supportive factor expression	(82, 83)
	Human immunodeficiency virus (human)	*In vitro* cultures of BMECs and mesenchymal stromal cells	Loss of HSPC supportive capacity—potential cause of pancytopenia	Direct infection, decrease of hematopoietic supportive factor expression	(84–86)
	Cytomegalovirus (human)	*In vitro* cultures of mesenchymal stromal cells	Loss of HSPC supportive capacity—potential contribution to BM failure posttransplantation	Direct infection, decrease of hematopoietic supportive factor expression	(87–89)

First, direct stimulation by microbial products and/or inflammatory cytokines has been reported to lead to profound changes in the transcriptional signature and signaling repertoire of components of the stromal microenvironment, which ultimately result in the diversion of hematopoietic output toward infection-induced patterns. Second, *via* the action of cytokines, microbial-derived peptides, or even through direct infection of specific stromal cell populations, pathogens can trigger detrimental effects in stromal cell integrity, leading to apoptosis or induction of aberrant differentiation programs. The resulting lesions in stromal networks will inevitably impact the normal regulatory features of the BM microenvironment. Finally, infections may drive defective stromal cell physiology by indirect mechanisms. For instance, massive consumption and depletion of inflammatory hematopoietic cells from the BM result in a severely altered cellular landscape, which may indirectly lead to deranged stromal cell maintenance/survival. Obviously, single pathogenic infections likely hinder BM stromal function at multiple levels and through the complex combination of several of these described modes of interference, which are non-mutually exclusive. Experimental data supporting the infection-mediated activation of the referred mechanisms of alteration of BM stromal cell physiology are discussed in detail in the following sections.

### Infection-Induced Remodeling of BM Stromal Cell Function

Akin to mature hematopoietic cells and HSPCs, certain BM stromal subsets have been shown to express specific receptors that render them susceptible to the direct action of microbial agents and inflammatory cytokines, as well as the molecular machinery needed to couple these signals into a defined intracellular activation program that alters their regulatory function. For instance, endothelial cells isolated from multiple organs, including the BM, consistently express TLR4 and its intracellular signaling protein, myeloid differentiation gene 88 (Myd88) (93). The integrity of this pathway in vascular cells is crucial for emergency granulopoiesis in the context of infections with Gram-negative bacteria. Using cell subset-specific deletion of Myd88 expression in mice, Boettcher et al. demonstrated that upon lipopolysaccharide (LPS)-mediated TLR4 stimulation, endothelial cells upregulate the principal granulopoietic cytokine, G-CSF to boost myeloid differentiation of hematopoietic progenitors (77). Although the specific contribution of BMECs with respect to those in vascular compartments of other organs was not determined, upregulation of G-CSF expression was most prominent in BMECs, suggesting the existence of at least a partial contribution of the local vascular compartment to overproduction of myeloid cells (90).

Albeit through different mechanisms, certain viral infections have been reported to similarly induce a skewing toward myeloid differentiation already at the level of HSPCs, through modulation of stromal-derived signals. *Lymphocytic choriomeningitis* virus (LCMV) is a natural murine pathogen, which has been widely employed in the discovery of general features of antiviral immune responses (94). During the early phases of acute LCMV infections, a myeloid HSPC fate is transiently enforced in order to accelerate production of myelomonocytic innate immune cells. The reactive shift in HSPC fate was shown to depend on the upregulation of IL-6 secretion by CAR cells, which in turn was triggered by IFNγ production by activated BM CD8 cytotoxic T cells (81). Of importance, previous studies had shown that acute LCMV infections induced transient BM aplasia, suppression of HSC function, and a pancytopenic state (95, 96). These effects were mediated, at least partially by the direct action of IFNα/β and IFNγ on hematopoietic cells. However, the possible contribution of stromal alterations in the context of LCMV infections had not been addressed in these studies. Notably, increased IL-6 production of CAR cells has also been observed upon infection with the rodent parasite *Toxoplasma gondii* (74). In this setting, induction of IL-6 not only augmented myeloid differentiation but also additionally elicited an anemic condition by stalling early stages of erythroid development in infected mice. Nevertheless, unlike LCMV infections, both IL-6 upregulation and anemia in this case were found to be independent of IFNγ, suggesting that multiple distinct mechanisms can operate to trigger local IL-6 secretion during different pathogenic inflammatory conditions (74).

Beyond altering the cytokine and growth factor milieu of the BM to instruct hematopoietic differentiation, infections or immunepathological reactions bring about (i) major changes in pro-migratory and retention cues provided by mesenchymal stromal cells and (ii) a breach in the barrier function and cell trafficking control exerted by BM vascular structures. Both effects critically contribute to the rapid mobilization of mature and immature hematopoietic cells from the BM to the periphery, which is a hallmark and a stereotypic host response to multiple pathogenic challenges (97–99). On the one hand, rapid deployment of mature cells, mostly of the myelomonocytic pool, increases the availability of circulating cells, which can be recruited to infected tissues for pathogen clearance (13, 14). In addition, mobilization of immature progenitors and HSCs leads to the transient establishment of extramedullary hematopoiesis (EMH) in other organs, mostly the spleen. EMH is a sign of dysfunction of the BM microenvironment, which is not only observed during infections but also in multiple stress-related conditions, and it is hypothesized to temporarily preserve early hematopoietic progenitors in a facultative supportive microenvironment (100, 101). However, a minor fraction of circulating HSPCs is found to traffic to peripheral tissues and differentiate *in situ* into immune cells, thereby contributing to local infectious control (102).

The mechanisms driving massive cellular exit from BM during infections are incompletely understood and will likely differ between the hematopoietic cell types mobilized and the type of pathogen. A general mechanism involves the interference of the CXCR4/CXCL12 axis, which is the major molecular pathway involved in BM retention described thus far. Expression and protein levels of CXCL12 in the BM are substantially reduced during certain bacterial infections, thus leading to release of HSPCs to peripheral circulation and lodging in spleen (76, 103). Given that CAR cells are the main source of CXCL12 in the BM and prominently express other adhesion molecules such as VCAM-1, it is highly likely that cell mobilization at least partially results from the erosion of the pro-retention function of this cell type. Supporting this notion, a recent study demonstrated that HSPC mobilization during *Escherichia coli* infection was mediated by increased G-CSF production, which has been shown to specifically reduce CXCL12 expression in CAR cells (104). Interestingly, in this infectious model, G-CSF secretion is triggered by the combined activation of TLR4 and NOD receptors in an undefined non-hematopoietic cell type, most likely endothelial as suggested by previous studies (76, 90). The attenuation of CXCL12-mediated retention signals does not only induce release of HSPCs from BM but also is hypothesized to contribute to rapid neutrophil egress during infections (105).

Besides this general mechanism, CAR cells additionally participate in BM cellular egress during infections in an active manner. Upon sensing of low levels of circulating LPS, BM perisinusoidal CAR cells upregulate expression of the pro-migratory chemokine CCL2, which is essential for emigration of CCR2^+^ inflammatory monocytes across sinusoidal vessels into systemic circulation (78). The resulting increase in frequencies of circulating monocytes was shown to be critical for efficient clearance of pathogen clearance of *Listeria monocytogenes* infections.

### Infection-Induced Damage to BM Stromal Cells

As observed for vascular and mesenchymal scaffolds in other lymphoid organs, pathogenic insults have been recently reported to selectively impact BM stromal cell survival (23, 106). Resulting cellular lesions can lead to imbalance in stromal composition and/or structural defects, which in turn have sizeable and distinct effects in the hematopoietic system. Deleterious actions in stroma may be induced by sharp increases in local pro-inflammatory cytokine levels. Using a murine model of sepsis induced by cecal ligation and puncture, Terashima and colleagues recently proved that mature, bone-lining osteoblast populations are strongly decreased during polymicrobial infection (75). Partial depletion of the osteoblast pool resulted in a paucity of IL-7 production in the BM microenvironment and consequently, a major loss of common lymphoid progenitors, which compromised T and B lymphopoiesis. In this case, the observed reduction in osteoblast numbers was independent of TLR signaling, but again at least partially mediated by abnormally high G-SCF levels (75). These observations are well in line with the previously reported negative effect of exogenously administered G-CSF on osteoblastic populations (104). Remarkably, infection-induced depletion of osteoblasts and its consequences could be mitigated through activation of osteoblastic proliferation using parathyroid hormone (75). Although, it should be cautioned that an IL-7-expressing niche in human BM tissues has not been described to date, these findings are highly relevant as they may contribute to mechanistically explain and potentially prevent the persistent lymphopenic state that frequently leads to fatal secondary infections in septic patients. Notably, an independent study using a similar model of polymicrobial systemic infection further revealed conspicuous signs of mesenchymal stromal cell damage in BM, which included a partial loss of structural integrity and the abundant emergence of adipocytes, indicative of the induction of aberrant differentiation programs in mesenchymal progenitor cells (107).

Bone marrow stromal cell subsets are also sensitive to the direct action of pathogenic virulence factors. For instance, administration of the bacterial second messenger bis-(3′–5′)-cyclic dimeric guanosine (c-di-GMP) was recently shown to strongly reduce endothelial and CAR cell numbers in BM and compromise their ability to produce relevant HSPC niche factors, such as CXCL12 or kit-L (80). Consequently, HSPCs were mobilized to the periphery, subsequently homing and proliferating in the spleen. c-di-GMP is known to trigger immune responses in a variety of cell types through the activation of the endogenous endoplasmic receptor stimulator of IFN genes (STING). Of importance, multiple cell types, including HSPCs and immune cells, express STING receptors. Hence, it cannot be ruled out that the damaging effect of c-di-GMP is at least to some extent mediated indirectly, through activation of inflammatory cascades in other cell types.

Finally, evidence suggests that stromal populations can be directly infected by certain intracellular pathogens this potentially leading to stromal cell death or dysfunction. Such is the case for viral agents, such as cytomegalovirus (CMV) and human immunodeficiency virus (HIV), which infect BM mesenchymal stromal cells and suppress their hematopoietic supportive capacity *in vitro* (see discussion below). In addition, it was recently reported that *Mycobacterium tuberculosis* displays specific tropism for the mesenchymal compartment of the BM, which acts as a reservoir for pathogen persistence and dissemination (79). However, from this study, it was unclear whether long-term latency in mesenchymal stromal cells had an impact in the bone forming or hematopoietic regulatory functions of MSCs.

### Deregulation of Hematopoietic Cell Crosstalk with BM Stroma

While much work has focused in understanding how stromal cells influence the BM hematopoietic compartment, regulatory crosstalk is most likely bidirectional and significantly less is known on the potential mechanisms by which hematopoietic cells may shape their microenvironment. Understanding such reciprocal modulation may become especially crucial during inflammatory stress situations in which the hematopoietic content of the BM undergoes drastic qualitative and quantitative alterations due to massive egress and entry of immune cell subsets, as well as biased HSPC lineage differentiation.

In steady state, myeloid cells have been reported to regulate CAR cell functionality at least *via* two different mechanisms. BM macrophages provide cues that ensure production of sufficient levels of CXCL12 by CAR cells both *in vitro* and *in vivo*, through a yet unknown mechanism (91). Depletion of CD169^+^ macrophage populations consequently results in abnormally low CXCL12 levels and defective HSPC retention in BM. Macrophages have been further reported to play a completely unanticipated role in the indirect modulation of HSPC mobilization *via* a complex mechanism involving CAR cells, as well as neutrophils. Macrophages are responsible of the clearance of aged, dysfunctional circulating neutrophils, which takes place among other organs in the BM (108). For their elimination, as neutrophils age they are recruited to the BM parenchyma in waves, which follow circadian fluctuations and are thus synchronized by sympathetic nervous signals. The time window of maximal BM neutrophil entry within the regular light–dark cycles was observed to precede that of most active HSPC egress to circulation. Mechanistically, it was demonstrated that BM macrophages engulfing aged neutrophils are activated through cholesterol receptors and relay a signal to CAR cells that temporarily downmodulates their CXCL12 expression (92). As a consequence, waves of HSPC mobilization toward the blood are rhythmically generated. Collectively, the findings described above strongly suggest that the changing dynamics of BM-resident myelomonocytic cell subsets in the context of infections could potentially affect BM stromal function, a hypothesis that deserves further in-depth studies. On the basis of the evidence discussed above, it seems clear that the BM microenvironment and each of its individual components represent highly susceptible substrates for the indirect action of pathogenic stimuli in the hematopoietic system.

## Clinical-Based Evidence of BM Stromal Dysfunction in Human Infections

Beyond the experimental studies in animal models here described, relevant information on the effects of pathogenic challenges on the BM microenvironment has been derived from the extensive clinical characterization of hematological syndromes induced by infectious diseases in humans. BM manifestations are associated to multiple pathogenic infections, especially by certain viruses, which frequently lead to pancytopenia, aplastic anemia, and/or severe failure of BM function (109, 110). In some cases, the pathophysiological mechanisms underlying BM symptoms and the nature of the cells affected have been identified. For instance, parvovirus B19 is well known to induce anemia through preferential infection of erythroid progenitors to halt their maturation (20, 109). In many infections, histological examination of BM biopsies reveals substantial microarchitectural damage, thus suggesting some sort of perturbation of stromal integrity (111). Yet, distinct stromal deficiencies in the context of infections have only been investigated in some detail for few human viral agents.

Infection with human CMV, a member of the Herpesvirus family, remains to this day one of the major complications following allogeneic BM transplants, eventually causing hematopoietic and graft failure, as well as fatal multi-organ disease (112). CMV infections, which are prevalent and remain latent in the majority of the population, are reactivated in immunosuppressed individuals. Evidence from human and murine studies strongly suggests that a major contributing cause lies in the direct damage that CMV inflicts to BM stroma. *In vitro*, CMV infects human BM mesenchymal stromal cultures compromising their hematopoietic supportive capacity (87–89). Likewise, murine CMV induces transient BM aplasia and has been shown to also infect BM mesenchymal cultures altering their capacity to secrete multiple growth factors and cytokines relevant for their control of hematopoiesis (82, 83). Similar *in vitro* observations have been made for other human and mouse viruses, such as hepatitis B virus and LCMV (113). Of interest, LCMV infections also impaired hematopoietic repopulation post-BM transplantation in mouse models (114). However, given that these viruses not only target stromal cells but also infect BM-mononuclear cells and directly suppress colony-forming capacity *in vitro*, it is conceivable that perturbations of both non-hematopoietic and hematopoietic cells will synergize to account for the observed failure in marrow engraftment (87, 94, 115).

A variety of hematopoietic disorders, including cytopenias and prominent BM morphological alterations, have also been extensively reported in HIV patients (116, 117). The etiology of hematopoietic BM syndromes in HIV-infected individuals remains hard to precise, given that besides the viral agent, multiple factors can contribute to the pathophysiology of BM impairment. These include drug-induced cytotoxicity, chronic inflammatory disease, the presence of additional opportunistic infections, and the development of disease-related neoplasms. Nonetheless, experimental evidence points to a direct effect of HIV infections in the BM stromal microenvironment. Early studies after the emergence of AIDS demonstrated that HIV infection was not only restricted to lymphocytic cells but also affected mesenchymal and endothelial cells in the BM (84–86). Upon infection, *in vitro* cultured mesenchymal stromal cells display suppressed hematopoietic supportive capacity and have been shown to become senescent and modify their differentiation potential when exposed to the viral proteins Tat and Nef (118).

## Concluding Remarks

Our understanding of the composition and organization of the cellular landscape of the BM has dramatically enhanced in the last decade. As progress has been attained, stromal cells that form the BM microenvironment have taken center stage, revealing as a compartment of unanticipated heterogeneity, versatility, dynamic nature, and pleiotropic functions. The phenotype, localization, and function of different stromal elements in the control of hematopoietic function continue to be explored. Nonetheless, with the knowledge generated thus far, new, highly pertinent questions arise on the role of stroma in stress situations in which an enhanced cellular supply is required or in which BM function is compromised. Indeed, the potential contribution of the BM microenvironment to pathological hematopoiesis has become increasingly apparent and the focus of multiple studies. Although much is still to be learnt, it seems clear that BM stromal cells respond in distinct ways to different microbial infections and the inflammatory responses launched by host organisms. On the one hand, stromal cell activation is likely an essential facet of the preprogrammed and coordinated biological response to dynamically adjust hematopoietic output to new demands. However, it also seems evident that acute and chronic inflammatory processes as well as direct microbial infection can have dire effects in stromal functionality that contribute to subsequent pathological hematopoiesis and possibly immune suppression. Hence, there is a clear need for refined knowledge on (i) the specific cellular subsets targeted during different pathogenic processes, (ii) the molecular cascades affected, (iii) the modes in which stromal changes may dictate reactive hematopoiesis, and (iv) the relative contribution of these changes to the global response in BM hematopoiesis compared to direct effects on hematopoietic cells. As hinted by existing studies (75), such information should directly translate into novel pharmacological strategies to prevent BM stromal cell damage and therefore therapeutically ameliorate hematologic symptoms caused by infections.

Nonetheless, the implications of such basic insights will certainly reach beyond the prevention of infection-associated hematopoietic alterations. It will be essential to understand whether ongoing low-grade persistent infections or sequels left in BM stroma by prior infectious events contribute to precipitate, if not directly drive, the development of more severe hematopoietic diseases. For instance, inflammation-driven functional remodeling of BM stromal components is critical for the development and progression of certain BM neoplasms (119). Hence, it can be speculated that preexisting inflammatory lesions in stromal cells could be relevant predisposing factors for hematologic neoplasms by providing a fertile ground for growth of malignant cells. Moreover, it will be crucial to determine whether and how infection-related perturbations may hinder the natural regenerative capacity of the BM stromal infrastructure, which is needed for the recovery of tissue integrity and function after myeloablative treatments widely employed nowadays. The fact that CMV infections still represent severe problems in BM transplant patients clearly exemplifies the need for more in-depth biological studies to uncover the pathophysiology of these processes. Finally, given the well-established link between inflammation and aging, it is tempting to speculate that the accumulation throughout an entire lifetime of unresolved injuries in BM stroma may cause, at least partially, the aged-associated defects in BM microenvironmental regulation of hematopoiesis (120). We are convinced that the use of currently available experimental models and technologies will help to provide clear answers to these and other questions on the relevance of stromal-derived signals for pathological hematopoiesis.

## Author Contributions

CN-A conceived and wrote the manuscript and prepared the figures. SI wrote the manuscript and prepared the figures.

## Conflict of Interest Statement

The authors declare that the research was conducted in the absence of any commercial or financial relationships that could be construed as a potential conflict of interest.
